# Satellite Glial Cells of the Dorsal Root Ganglion: A New “Guest/Physiopathological Target” in ALS

**DOI:** 10.3389/fnagi.2020.595751

**Published:** 2020-11-09

**Authors:** María Ruiz-Soto, Javier Riancho, Olga Tapia, Miguel Lafarga, María T. Berciano

**Affiliations:** ^1^Department of Anatomy and Cell Biology, University of Cantabria, Santander, Spain; ^2^“Centro de Investigación Biomédica en Red sobre Enfermedades Neurodegenerativas” (CIBERNED), Madrid, Spain; ^3^“Instituto de Investigación Sanitaria Valdecilla” (IDIVAL), Santander, Spain; ^4^Service of Neurology, Hospital Sierrallana, Torrelavega, Spain; ^5^Department of Medicine and Psychiatry, University of Cantabria, Santander, Spain; ^6^“Universidad Europea del Atlántico”, Santander, Spain; ^7^Department of Molecular Biology, University of Cantabria, Santander, Spain

**Keywords:** ALS (Amyotrophic lateral sclerosis), satellite glial cells (SGCs), sensory, SOD1 mouse G93A, glia

## Abstract

**Introduction:** Amyotrophic lateral sclerosis (ALS) might not only be circumscribed to the motor system but also involves other neuronal systems including sensory abnormalities. In line with this notion, we aimed to assess the pathophysiology of sensory disturbances in the SOD1^G93A^ mouse model of ALS, focusing on the satellite glial cells (SGCs) at the dorsal root ganglion (DRG) as a new potential target of the disease.

**Material and Methods:** The presence of sensory disturbances was evaluated using von Frey, hot plate, and hot water tail immersion tests at 75 days old, which represented the motor-pre-symptomatic stage. Cell biology analysis was performed at 75 and 95 days old and included conventional histology, immunofluorescence, and electron microscopy of sensory neuron-SGC unit dissociates as a well as western blotting from DRG lysates.

**Results:** At 75 days old, von Frey and hot plate tests demonstrated clear thermoalgesic disturbances in ALS transgenic mice. Histological studies of the SN-SGC units revealed abnormal SOD1 accumulation, which was associated with nitro-oxidative stress and biogenesis of lipid droplets in SGCs. Interestingly, these alterations led to a progressive lysosomal storage disorder and occasionally vacuolar degeneration in SGCs.

**Conclusions:** SGCs emerge as a primary pathophysiological target in the SOD1 transgenic murine model of ALS, clearly reinforcing the pathogenic role of glial cells in motor neuron disease. Presymptomatic alterations of SGCs, might not only be responsible of sensory disturbances in ALS, but due to spinal cord sensory-motor circuits could also contribute to anterior horn motor disturbances.

## Introduction

Amyotrophic lateral sclerosis (ALS) is the most common neurodegenerative disease affecting motor neurons (MNs) with an annual incidence that ranges from 1 to 3 cases per 100,000 individuals (Logroscino et al., 2010; Riancho et al., 2016). The pathogenesis of ALS has not yet been completely elucidated. A small percentage (20%) of cases have a familial origin (fALS) that is related to mutations in specific causative genes such as SOD1, CR9ORF72, TARDBP, and FUS (for a review, see Zufiría et al., 2016). In contrast, the vast majority of cases are thought to be sporadic (sALS) and caused by interactions between genes and environmental conditions, leading to disease onset in genetically predisposed individuals (Riancho et al., 2018). Clinically, ALS is characterized by the progressive degeneration of both upper and lower MNs typically results in progressive muscle wasting and usually lead to death within 3 years after symptom onset (Amato and Russell, 2008). Since its description, ALS has been classically considered as a “motor system-circumscribed disease”; however, other clinical manifestations, including autonomic disorders, cognitive impairment and sensory disturbances, have been reported in patients with ALS. Regarding the latter, several experimental and clinical studies support some degree of sensory system impairment in individuals with ALS (Chiò et al., 2017; Riancho et al., 2020b).

Concerning preclinical studies, most evidence has been generated using the murine SOD1^G93A^ model. First, Guo et al. (2009) used this model to study the sensory system at several levels, including the dorsal roots, dorsal root ganglia (DRG), and posterior column tracts. Interestingly, the authors observed a loss of axons, cellular alterations, and demyelination. Shortly thereafter, Sábado et al. (2014) reported demyelination and a loss of axons in the posterior roots as well as loss of neurons and SOD1 accumulation in the DRG of this murine model. Consistent with this finding, researchers have postulated that sensory disorders in the SOD1 mutant murine model might be associated with the accumulation of a neurotoxic splice variant of peripherin (Sassone et al., 2016). Within this model, some investigators have noted that proprioceptive sensory neurons are particularly susceptible to pathology (Sábado et al., 2014; Seki et al., 2019). In addition, according to Vaughan et al. (2018) cultured sensory neurons harboring mutations in either TDP43 and SOD1 exhibit lower growth rates, reduced neurite branch generation, and an increased susceptibility to cellular stress, thus suggesting important roles for this neuronal population in ALS-related pathogenesis.

The DRG contains the cell bodies of neurons that transmit the sensory information (proprioceptive, light touch, vibration, thermoceptive, and nociceptive information) from the periphery to the central nervous system (CNS) through the dorsal and anterolateral tracts of the spinal cord (Haberberger et al., 2019). In addition to controlling sensory information, proprioceptive sensory neurons are key modulators of motor behavior that integrate the sensory and motor systems into the CNS. Interestingly, sensory neurons have been recently suggested as ALS targets in a *Drosophila* model of MN disease (Held et al., 2019).

Classically, rodent DRG neurons are classified into three main morphological based on their size and the distribution of their organelles: A (large), B (medium size), and C (small dark) (Pena et al., 2001). Type A neurons have thick myelinated fibers and are mainly mechanoreceptive (proprioceptive). Type B neurons exhibit thin myelinated fibers and are mechanoreceptive and nociceptive. Finally, non-myelinated, slow conducting, type C neurons are mainly involved in thermo- and nociception (Wotherspoon and Priestley, 1999; Hunt and Koltzenburg, 2005; Berta et al., 2017). Each sensory neuron (SN) is wrapped by the cell bodies and laminar processes of several satellite glial cells (SGCs), forming a morphological, and functional unit (SN-SGC unit) (Pannese, 1981; Hanani, 2005). SGCs play an important role in regulating sensory neuron function, particularly in controlling the neuronal microenvironment (Hanani, 2005; Haberberger et al., 2019).

Although the selective cell death of MNs is a key feature of ALS, other tissues, and organs may be responsible for the clinical manifestations of the disease (for a review, see Zufiría et al., 2016). In fact, as shown in our recent study, that dermal fibroblasts from patients with ALS recapitulate alterations typical of ALS motor neurons and exhibit an abnormal DNA-damage response (Riancho et al., 2020a). Therefore, the DRG in the PNS might contribute not only to the subtle sensory manifestations of patients with ALS but also to MN degeneration by impairing sensory-motor spinal networks.

The aim of this study is to determine the cellular basis of the sensory system impairment in SN-SGC units in SOD1^G93A^ mice. To date, the SOD1 transgenic mouse has been the most extensively employed animal model for preclinical investigations of ALS. High-copy SOD1^G93A^ transgenic mice recapitulate an important proportion of the pathophysiological features of human ALS, including progressive MN degeneration and neuromuscular function loss as well as a reduced lifespan (Gurney et al., 1994). Although this mouse model is based on a familial form of the disease, several authors have highlighted its translational value in studies of sALS (Bosco et al., 2010). The potential contribution of SGCs to the sensory component of ALS at both, late presymptomatic (75 days old) (Riancho et al., 2015) and symptomatic (95 days old) stages should receive special attention. Based on our results thermonociceptive and fine touch dysfunction accompanied motor alterations in the SOD1^G93A^ mouse model of ALS. Sensory disturbances correlated with high expression of mutant SOD1 in SGCs, which appeared to increase lipid peroxidation and an aberrant storage of lysosome-related structures, preferentially in SGCs belonging to type B and C units.

## Methods

### Animals

The transgenic mice used were B6SJL-Tg (SOD1^G93A^ 1Gur/J (SOD1^G93A^) obtained from The Jackson Laboratory (Bar Harbor, ME, USA) and maintained at the Animal Service of the University of Cantabria. The colony was maintained by mating heterozygous transgenic males with B6SJLF1/J hybrid females. Real time quantitative PCR of DNA obtained from tail tissue was used for genotyping, with specific primers detecting human SOD1 and the housekeeping mouse gene ApoB. Primer sequences were: SOD1: GGG AAG CTG TTG TCC CAA G and CAA GGG GAG GTA AAA GAG AGC; ApoB: TCA CCA GTC ATT TCT GCC TTT G and GGG AAG CTG TTG TCC CAA G. Transgenic mice and control littermates were housed under controlled temperature and humidity, with a 12-h light/dark cycle and free access to water and food. The experimental protocol was approved by the Ethics Committee of the University of Cantabria following the Spanish legislation. All animal experiments were carried out in accordance with the EU Directive 2010/63/EU.

Male animals were distributed into four groups: two wild-type mice (75 and 95 days old) and two SOD1^G93A^ mice (75 and 95 days old). In SOD1^G93A^ mice these age stages correspond to the presymptomatic and symptomatic stages of the disease in this murine model. Globally, sensory tests included 23 transgenic SOD1 and 23 wildtype mice, respectively. For light microscopy and immunofluorescence 16 control and 16 SOD1 animals were used. Finally, biochemical analysis was performed in 3 transgenic and 3 wild type mice, respectively.

### Rotarod and Sensory Tests

For investigating the presence of subjacent sensory disorders and in order to not be biased by motor symptomatology, tests were performed on day 75 of life. Globally 23 wild-type and 23 transgenic mice were used. Before assessing sensory disturbances mice weight and rotarod performance and were evaluated as previously described (Riancho et al., 2015). Sensory tests included von Frey hair test, hot water tail immersion test, and hot plate test. These tests assess nociception induced by both, mechanical and thermal stimuli.

For von Frey test, mice were placed into the corresponding testing area and left there for 20 min. Once habituated, mechanical stimuli with von Frey filaments (Semmes Weinstein von Frey Aesthesiometer for Touch Assessment, Stoelting Co, Illinois EEUU) were applied on the forelimb of each animal. Limb shaking or limb licking, were considered as positive responses. Nociceptive threshold was then determined as the force evoking a 50 percent of positive responses.

The hot water tail immersion test evaluated the latency of the tail flick reaction in mice after immersing their tails in a constant temperature bath. Three centimeters of mice tail were submerged in hot water at 45, 47, and 49°C, respectively. A cut off point of 60 s was stablished in order to avoid tissue damage. For hot plate test, mice were place into 2 metallic cylinders which had been previously heated up to 50 and 52°C. Then the latency until the animal exhibited a positive response, considered as limb shaking, limb licking, or jumping, was measured. A cut off point of 120 s was considered to evade mice injuries. All sensory tests were done in quintuplicate.

### Light Microscopy and Immunofluorescence

To study histological changes, 8 mice of each group (SOD1 and control mice) were euthanized on days 75 and 95, respectively, and their dorsal root ganglia (DRG) processed for light microscopy and immunofluorescence. After deep anesthesia with pentobarbital (50 mg/kg) mice were perfused with 3.7% paraformaldehyde in PBS (pH 7.4) for 15 min. DRG were dissected and post-fixed for 2 h. Small tissue fragments were processed for mechanical dissociation of SN-SGC units following the procedure of Pena et al. (2001). Briefly, DRG fragments were transferred to a drop of PBS on a siliconized slide. Then, a coverslip was applied on top of the slide and the tissue was squashed by percussion with a histologic needle to dissociate neuronal cell bodies. The preparation was then frozen in dry ice, and the coverslip removed using a razor blade. Using this procedure most SN-SGC units remained adhered to the slide. Cell samples were processed in 96% ethanol at 4°C for 10 min and rehydrated progressively in 70% ethanol and PBS. Some preparations were stained with propidium iodide (PI), a fluorescent staining of nucleic acids. For immunofluorescence, squash preparations were sequentially treated with 0.5% Triton X-100 in PBS, 0.1 M glycine in PBS containing 1% bovine serum albumin and incubated with the primary antibody overnight at 4°C. Then, the sections were incubated with the specific secondary antibody conjugated with FITC or Cy3 and mounted with Vectashield (Vector USA). Confocal images were obtained with a LSM510 (Zeiss, Germany) laser confocal microscope using the 63x oil (1.4 NA) objective. In order to avoid overlapping signals, images were obtained by sequential excitation at 488 and 543 nm, to detect FITC and Cy3, respectively. Images were processed using Photoshop software.

To determine the relative nuclear and cytoplasmic levels of SOD1 in both sensory neurons and SGCs in wild-type and SOD1^G93A^ mice confocal images were recorded by using a 63x oil (1.4 NA) objective and the same confocal settings at resolution of 1,024 × 1,024 pixels. Images were background corrected by reference regions outside the tissue and fluorescence intensities were estimated by using the ImageJ software (NIH, Bethesda, Maryland, USA; http://rsb.info.nih.gov/ij/). We use three animals per experimental group and at least 35 neurons per animal were sampled.

The following primary antibodies were used. Goat polyclonal antibody anti-cathepsin D (dilution 1:100; Santa Cruz Biotechnology, USA) and rabbit polyclonal antibodies anti-SOD1 (dilution 1:100; Enzo Life Sciences, Switzerland) and anti-ubiquitin-protein conjugates (dilution 1:50; Biomol International).

### *In situ* Determination of Lipid Peroxidation

For the determination of lipid peroxides squash preparations of DRG fixed with 3.7% paraformaldehyde in buffer phosphate 0.12 M were air dried, washed in PBS and incubated with the probe C11-BODIPY^581/591^ or BODIPY^493/503^ (Molecular Probes, USA) at the concentration of 1 μg/mL for 30 min at 37°C (Liu et al., 2015). Then, the samples were washed in PBS and mounted with the antifading agent Vectashield-DAPI (Vector Laboratories, USA). Images were acquired immediately with a Zeiss LSM 510 microscope.

### Electron Microscopy

For electron microscopy, wild-type and SOD1^G93A^ mice were deeply anesthetized and perfused with 1% glutaraldehyde and 1% paraformaldehyde in 0.12 M phosphate buffer. Tissue samples of the DRG were postfixed with 2% osmium tetroxide dehydrated in increased concentrations of ethanol and embedded in Araldite (Durcupan, Fluka, Switzerland). Semithin sections (1 mμ thick) were stained with toluidine blue for the light microscopy examination of the DRG. Ultrathin sections mounted in copper grids were stained with uranyl acetate and lead citrate and examined with a Jeol 2011 electron microscope operated at 80 kV.

### Western Blotting

For Western blot (WB) analysis, DRG were homogenized in a lysis buffer (0.1 mol/L NaCl, 0.01 mol/L Tris-HCl, pH 7.5, 1 mmol/L EDTA, and 1 μg 7 Ml aprotinin) and the homogenates were centrifuged. Overall, 3 SOD1 and 3 control mice were used, respectively. The protein in the supernatants were subjected to SDS-PAGE electrophoresis and afterwards transferred to polyvinylidene difluoride membranes, stained with antibodies and visualized with the Odyssey system (LI-COR Biotechnology). Alpha-Tubulin was used as protein loading control. The antibodies used were rabbit polyclonal anti-SOD1 (dilution 1:1,000; Enzo, Life Sciences, Switzerland), goat polyclonal antibody anti-cathepsin D (dilution 1:2,000; Santa Cruz Biotechnology, USA) and mouse monoclonal anti-Tubulin (dilution 1:000; Abcam, Cambridge, USA). ImageJ software (U.S. National Institutes of Health, Bethesda, Maryland, USA) was used to quantify the density and size of the blots.

### Statistical Analysis

The significance of the differences in weight differences was tested by unpaired Students'*t*-test, while non-parametric Mann-Whitney *U*-test was used to compare sensory test scores of wild-type and SOD1^G93A^ mice.

## Results

### Presymptomatic SOD1 Transgenic Mice Exhibit Sensory Disturbances at 75 Days of Age

**Figure 1:**
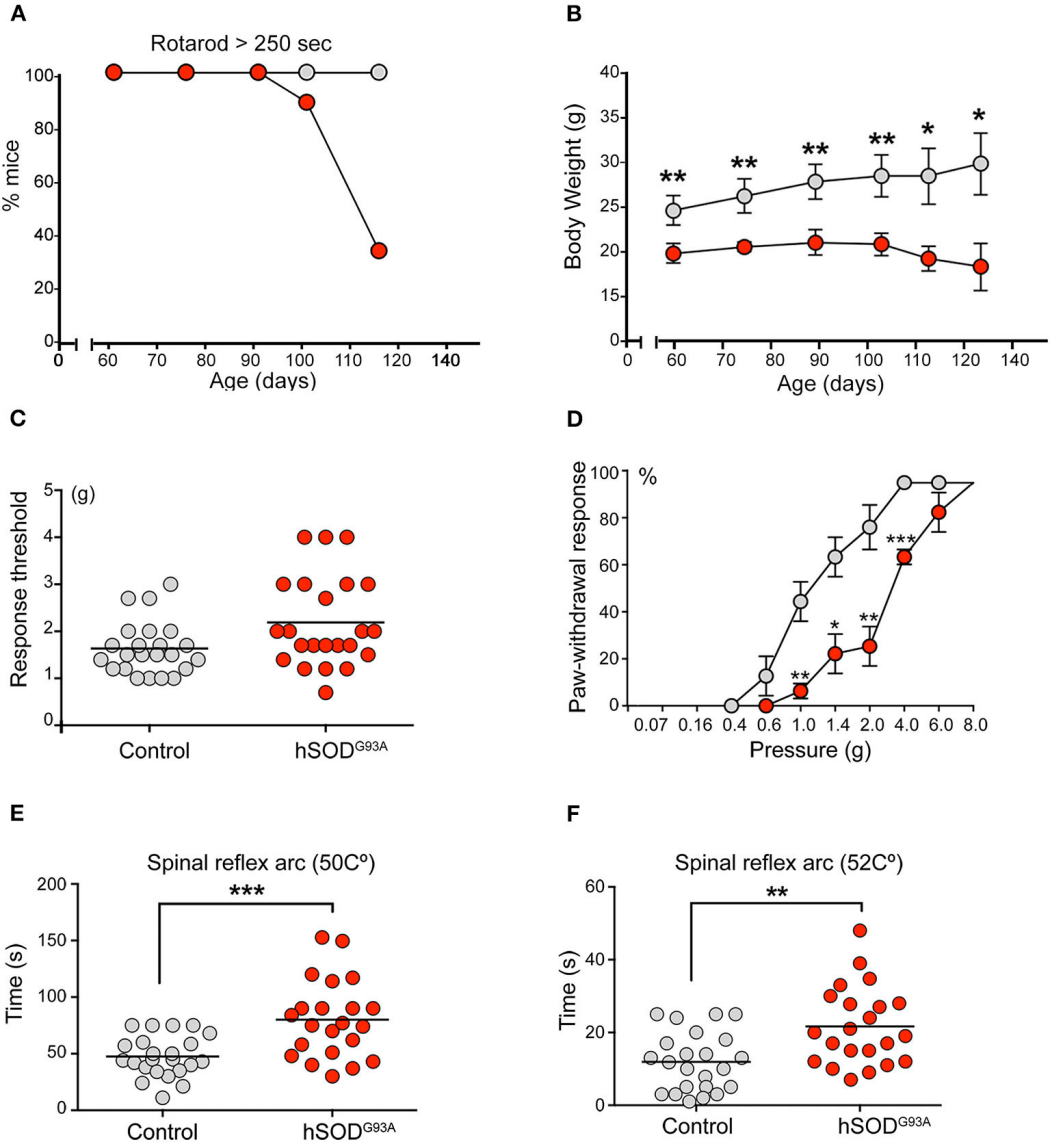
Sensitive pathology in SOD1^G93A^ mice. **(A)** Rotarod test showed a progressive neuromuscular deterioration in SOD1 transgenic mice from day 95 onwards. **(B)** ALS mice exhibited a significant lower weight during the whole experimental period (Students'*t*-test, *p < 0.05; **p < 0.005). **(C)** Globally, von Frey test evidenced a lower non-significant tactile threshold in the control group in comparison to SOD1 transgenic mice (1.65 vs. 2.14). Of note, 3 ALS transgenic mice showed a markedly higher tactile threshold, thus suggesting some degree of tactile disturbance. **(D)** When evaluating the proportion of positive responses depending on the filament force, a clear significant delayed response was evidenced in SOD1 transgenic mice from 1 g upwards (Students'*t*-test, *p < 0.05; **p < 0.005; ***p < 0.0005). **(E,F)** Hot plate test evidenced a significant delayed response in ALS transgenic mice when compared to control mice at both 50 Celsius degrees **(E)** 49 vs. 74 s; Students'*t*-test, *p < 0.05) and 52 Celsius degrees **(F)** (10 vs. 21 s; Students'*t*-test, **p < 0.005).

The presence of sensory alterations was evaluated in 75-day-old mice to avoid any motor-induced bias. Before the assessment, all mice performed the rotarod test to exclude any subjacent neuromuscular disorder. At that age, no differences in rotarod performance were observed between wild-type (hereafter referred to as control) and transgenic mice. As expected, a progressive worsening in rotarod performance was observed beginning at day 90 of life (Figure 1A). Consistent with our previous studies (Riancho et al., 2015), ALS transgenic mice exhibited a significantly lower weight than controls (Figure 1B).

The mechanical nociceptive threshold von Frey hair test evaluated the sensation of pain. This test is based on the principle that a compressed elastic column will buckle elastically at a specific constant force. The filaments (hairs) are used to provide a range of forces to the mouse forelimb and identify the force to which the animal reacts because it causes a painful sensation. Globally, the results of the von Frey test revealed a lower but non-significant tactile threshold (force at which the animal moved off its limb at least 50% of the time) in the control group than in ALS transgenic mice (mean force, 1.6 ± 0.21 vs. 2.1 ± 0.09, *p* = 0.0634, Figure 1C). Notably, 3 ALS transgenic mice exhibited markedly higher tactile thresholds, thus suggesting some degree of tactile disturbance. Although no significant differences were noted in the global threshold analysis, clear and significant delays were observed when forces of 1 g and greater were applied to the SOD1^G93A^ group, based on the percentage of positive responses to the filament force (Figure 1D). These results reveal a delayed tactile response in transgenic mice.

The hot water tail immersion test and the hot plate test were performed to assess temperature-related nociception. These tests are complementary. The former mainly evaluates the spinal reflex arc (König et al., 1996), whereas the latter requires supraspinal processing (Le Bars et al., 2001). Regarding the hot water tail immersion test, no significant differences in tail withdraw latencies were not observed between transgenic and control mice at 45, 47, and 49°C. The hot plate test, which evaluates the latency of a mouse to jump off a warming plate, was performed with plates at 50 and 52°C (Figures 1E,F). At both temperatures, SOD1 transgenic mice exhibited a significantly delayed latency (mean latency, 47.8 ± 2.53 vs. 65.2 ± 2.46 s at 50°C, *p* = 0.0217*;* 12.04 ± 0.86 vs. 20.6 ± 2.34 s at 52°C, *p* = 0.0024) (Figures 1E,F).

### SN-SGC Units Express Mutant SOD1 at High Levels in SOD1^G93A^ Mice

**Figure 2:**
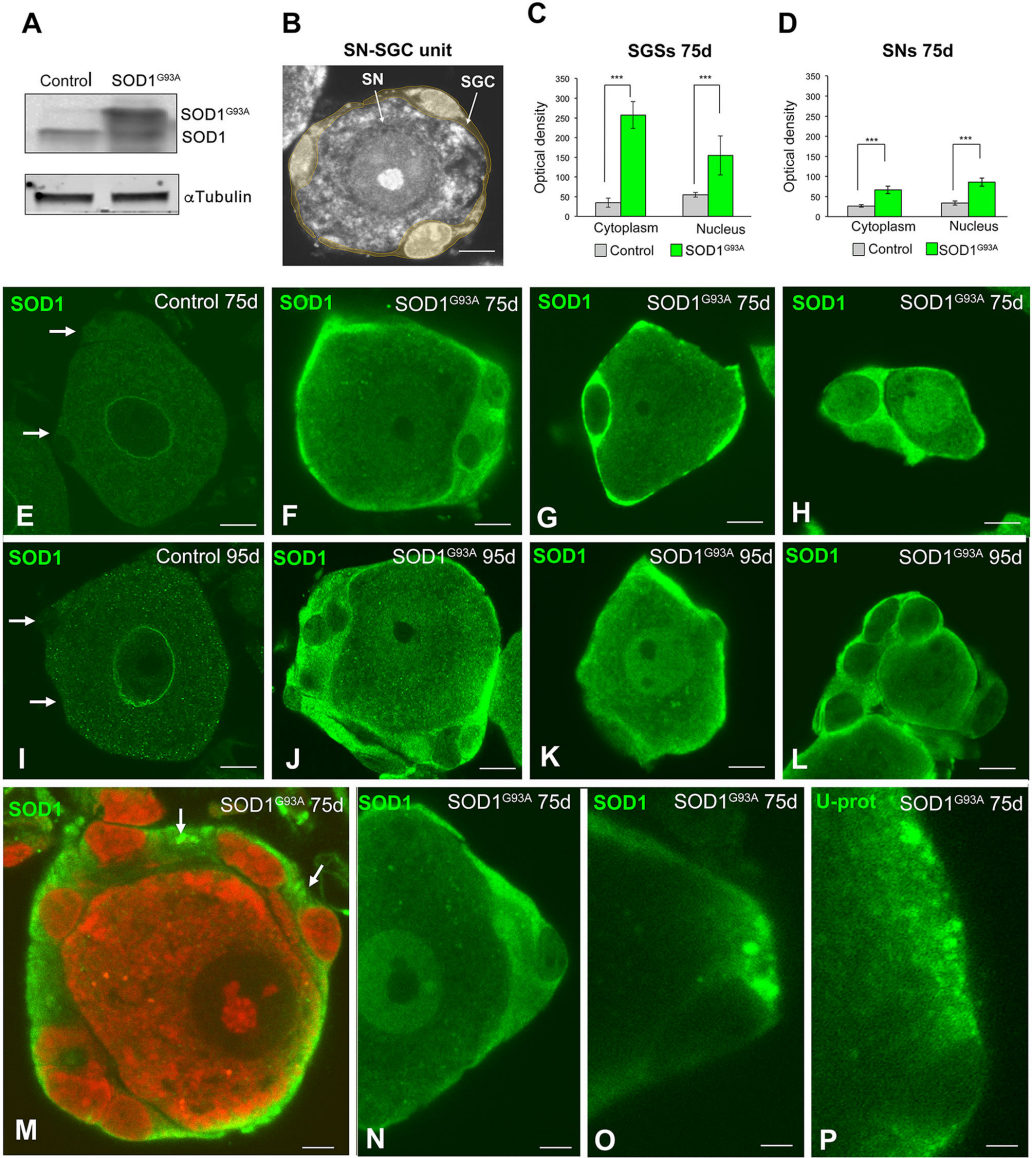
Expression of SOD1 in SN-SGC units from control and SOD1^G93^ mice. **(A)** Western blotting of SOD1 expression in DRG lysates from control and SOD1^G93A^ mice. Note the high levels of SOD1^G93A^ expression found in SOD1 transgenic mice. α-tubulin was used as load control. **(B)** Representative example of a typical SN-SGC unit in a control DRG. GSCs appear colored in pale yellow. **(C,D)** Densitometric analysis of SOD1 expression in the nucleus and cytoplasm of SGCs and sensory neurons at 75 and 95 days of age. **(E–L)** Immunodetection of SOD1 in control and SOD1^G93^ SN-SGC functional units at 75 **(E–H)** and 95 **(I–L)** days of age. **(E,I)** Images of control SN-SGC units showing low levels of SOD1 expression in the nucleus and cytoplasm from both sensory neuron and SGCs (arrows). **(F–H,G–L)** Representative examples of types **(A–C)** SN-SGC units from SOD1^G93A^ at 75 and 95 days of age. Note the high levels of cytoplasmic SOD1 in SGCs and the moderate expression of SOD1 in sensory neurons. **(M)** A SN-SGC unit of a SOD1^G93^ mouse immunolabeled for SOD1 and counterstained with PI illustrating the high concentration of SOD1 in the glial cytoplasm and its aggregation in cytoplasmic inclusions (arrows). Note the low expression of SOD1 in the neuronal cytoplasm and the well-preserved Nissl substance and prominent nucleolus counterstained with PI. **(N,O)** Detail of SGC cytoplasm from SOD^G93A^ mice showing areas with different SOD1 concentration and the presence of inclusions highly enriched in SOD1. **(P)** Immunodetection of ubiquitin-protein (Ub-prot) conjugates forming cytoplasmic aggregates in a SGC from a SOD1^G93^ mouse. The cell nucleus of the SGC appears unstained. Scale bar: **(B)** and **(E–L)**: 10 μm. **(M–P)**: 5 μm. Student *t*-test in **(C,D)** *p* < 0.001, ***p < 0.001.

The levels of both endogenous mouse SOD1 and mutant SOD1^G93A^ were estimated by performing Western blotting of DRG lysates using an antibody that recognizes murine and human SOD1. As expected, non-significant differences in murine SOD1 levels were detected in the DRG between control and SOD1^G93A^ mice. In contrast, high levels of SOD1^G93A^ were detected in the SOD1^G93A^ DRG (Figure 2A). This finding confirms the overexpression of the mutant human SOD1 in the DRG of transgenic SOD1^G93A^ mice and suggests that the high levels of the mutant human dismutase do not exert a negative dominant effect on wild-type murine SOD1 expression. Thus, the gain of function of the mutant SOD1^G93A^ rather than the loss of function of the murine SOD1, might be involved in the sensory dysfunction observed in SOD1^G93A^ mice.

Next, we investigated the subcellular distribution of SOD1 in SN-SGC units (Figure 2B) from control and SOD1^G93A^ mice by performing immunofluorescence staining using a polyclonal rabbit anti-SOD1 antibody. At 75 days of age, a very weak cytoplasmic signal was detected in both neurons and SGCs from control mice (Figure 2E). Conversely, in SN-SGC units from the SOD1^G93A^ mice, high SOD1 expression was observed in SGCs, and to a lesser extent, in sensory neurons (Figures 2F–H). Moreover, SOD1 expression was markedly increased in the cytoplasm of SGCs compared with the nucleus (Figures 2F–H). This observation was confirmed by the densitometry analysis of the fluorescent intensity of SOD1 in the nucleus and cytoplasm of SGCs, that revealed significant differences between these two compartments (Figure 2C). In sensory neurons from SOD1^G93A^ mice, SOD1 expression was generally increased in the nucleus compared with the cytoplasm (Figures 2D,H). At 95 days of age, a similar pattern of SOD1 expression was observed in SN-SGC units from both control and SOD1^G93A^ mice when compared to their respective 75-day-old control and SOD1^G93A^ mice (Figures 2I–L). Interestingly, some SGCs exhibited cytoplasmic SOD1 aggregates in SOD1^G93A^ mice (Figures 2M–O). Moreover, cytoplasmic aggregation of SOD1 was associated with the accumulation of ubiquitylated proteins as revealed by immunostaining for ubiquitin-protein conjugates (Figure 2P). Based on the biochemical determination of the levels of the SOD1 protein and the reduced expression of this dismutase in control SN-SGC units, the SOD1 immunofluorescence signal mainly corresponded to the transgenic expression of the mutant SOD1 protein.

### Overexpression of Mutant SOD1^G93A^ Is Associated With Nitro-Oxidative Stress and Biogenesis of Lipid Droplets in SGCs

**Figure 3:**
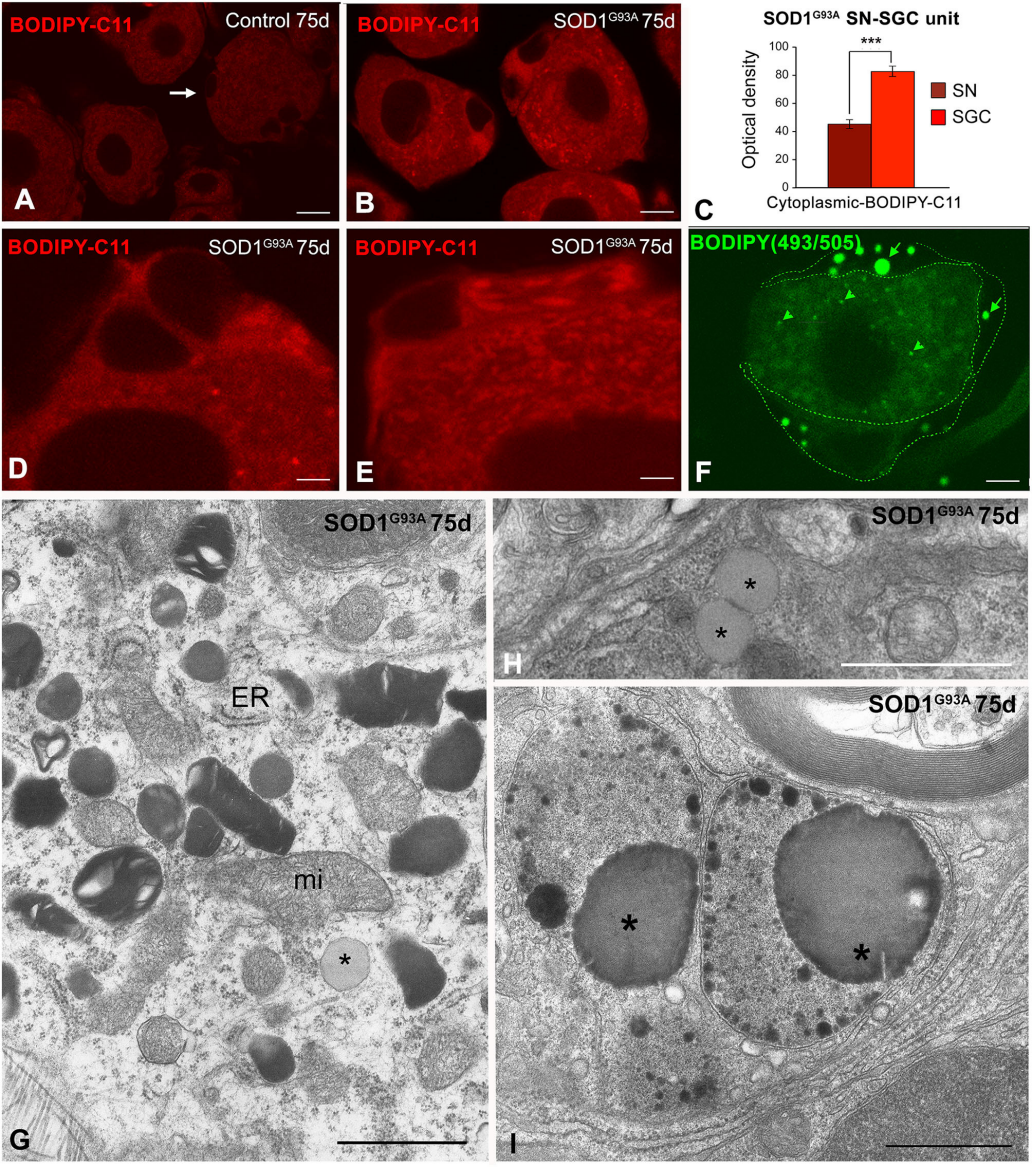
**(A–E)** *In situ* determination of lipid peroxidation with the BODIPY-C11 probe in SN-SGC units from control **(A)** and SOD1^G93^ mice **(B,D,E)**. **(A)** In control SN-SGC units both sensory neurons and SGCs (arrows) exhibit low fluorescent signal of lipid peroxidation in the cytoplasm. **(B,D,E)** Lipid peroxidation signal intensity notably increases in the cytoplasm of both sensory neurons and SGCs from SOD1^G93^ mice, although BODIPY-C11 labeling was substantially higher in SGCs. Note the absence of fluorescent signal in the nucleus. **(C)** Densitometric analysis of the cytoplasmic BOBIDY-C11 fluorescent signal intensity in sensory neurons and SGCs from SOD1^G93^ mouse at day 75 of age. **(F)** BODIPY-493/503 staining of a SN-SGC unit from the SOD1^G93^ mouse at day 75 of age. Spherical lipid droplets of neutral lipids appear intensity labeled with the probe. Lipid droplets are larger and more abundant in SGCs (arrows) than in the sensory neuron (arrowhead). The limits of the SGC have been drawn with dashed green lines. **(G)** Ultrastructural image of the SGC cytoplasm from a SOD1^G93A^ mouse illustrating the presence of numerous lysosomes with a heterogeneous morphology, isolated cisterns of endoplasmic reticulum (ER), some mitochondria (mi) and a lipid droplet (asterisk). **(H)** Detail of two spatially associated lipid droplets (asterisks) in a SGC from the SOD1^G93^ mouse. **(I)** Electron micrograph showing very complex lysosomal compartments that includes large lipid droplets (asterisks). Scale bar: **(A,B,F)** 10 μm; **(D,E)** 5 μm; **(G,H)** 1 μm; **(I)** 2 μm. Student *t*-test in **(C)** *p* < 0.001.

As an *in situ* marker of oxidative stress, we assessed lipid peroxidation levels using the fluorescent probe BODIPY-C11. The lipid peroxidation signal was clearly increased in dissociated SN-SGC units from SOD1^G93A^ mice compared with control animals (Figures 3A,B). Lipid peroxides accumulated in the cytoplasm of both sensory neurons and SGCs from SOD1^G93A^ mice and significantly increased levels were found in the latter (Figure 3B) as evidenced by the results of comparative analysis of the optical density of the fluorescent BODIPY-C11 signal (Figure 3C). Moreover, the cytoplasmic distribution of the BODIPY-C11 signal in SGCs was not homogeneous, and some cytoplasmic domains exhibited higher fluorescence intensity (Figures 3D,E), which may reflect either the formation of SOD1 aggregates or the existence of clusters of membrane-bound organelles with high levels of lipid peroxidation.

The cellular stress response of ROS and free radical generation may induce the formation of lipid droplets that accumulate as neutral lipid in non-adipocyte cells (Liu et al., 2015). We used the lipophilic fluorescent probe BODIOY-493/503 to assess whether mutant SOD1^G93A^ overexpression induced the formation of lipid droplets. Lipid droplets labeled with this probe were frequently detected in SGCs from the 75-day-old SOD1^G93A^ mice and to a lesser extent in sensory neurons but not in samples from control mice (Figure 3F). The electron microscopy analysis confirmed the presence of lipid droplets with a variable electron density in SOD1^G93A^ SN-SGC units, which appeared either as isolated lipid droplets surrounded by cytoplasmic organelles (Figures 3G,H, **6F**) or formed part of more complex lysosome-related structures (Figure 3I).

### Overexpression of Mutant SOD1^G93A^ Induces a Lysosomal Storage in SGCs

**Figure 4:**
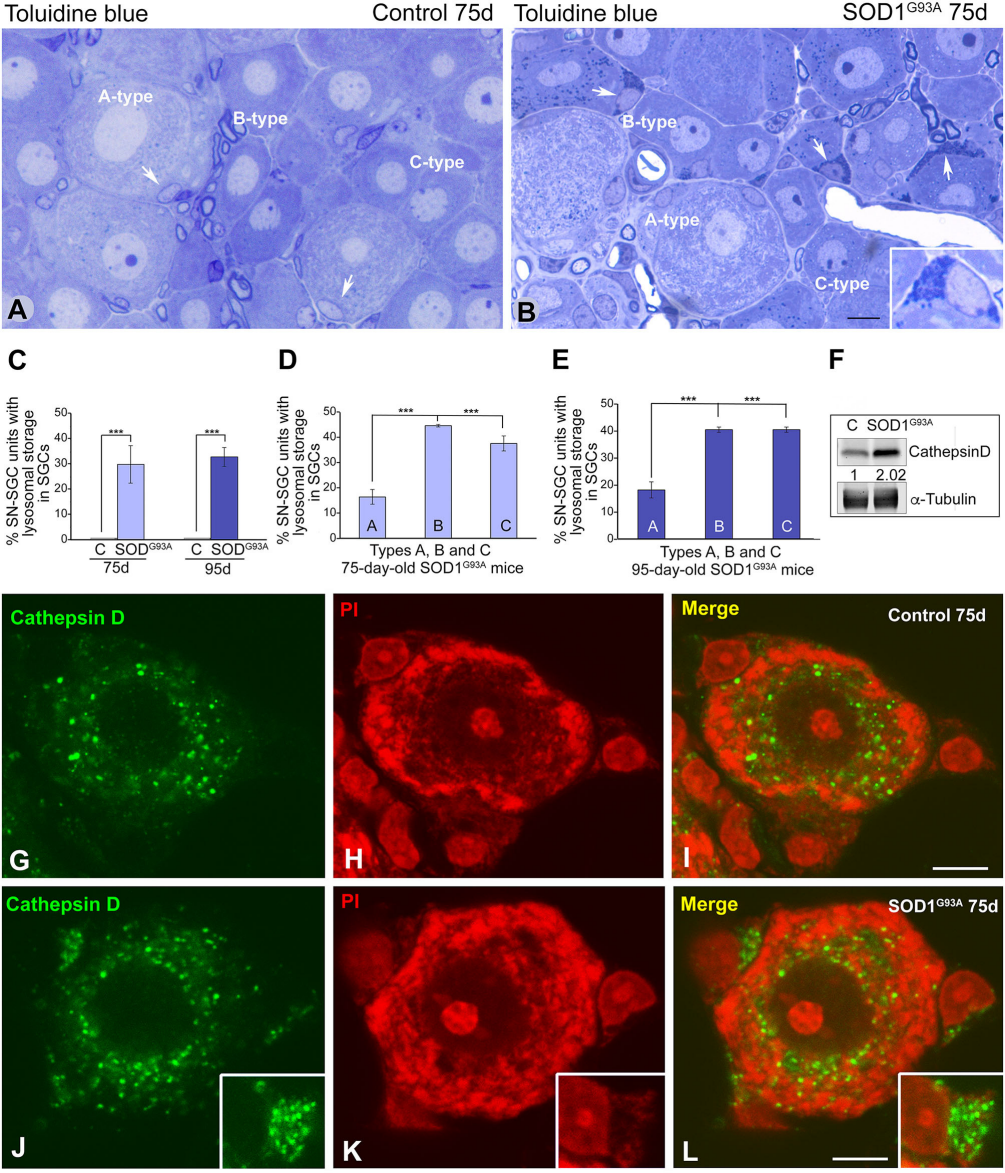
SGCs from SOD1^G93^ mice markedly increase the lysosomal compartment. **(A,B)** Semithin sections of DRG from control **(A)** and SOD1^G93^ mice **(B)** stained with toluidine blue illustrating the organization of SN-SGC units. Note the accumulation of basophilic granules identified as lysosomes in some SGCs from SOD1^G93^ mice (arrows and inset in **B**). **(C)** Quantitative analysis of SN-SGC units from control and SOD1^G93^ mice carrying basophilic granules in the SGC cytoplasm at 75 and 95 days of age. **(D,E)** Quantitative analysis of the proportion of types **(A–C)** SN-SGC units from SOD1^G93^ mice carrying basophilic granules in the SGC cytoplasm at 75 **(D)** and 95 **(E)** days of age. **(F)** Representative example of a western blot of cathepsin D expression in DRG from SOD1^G93^ and wild-type mice. Alpha-tubulin was used as loading control. Note the increased expression of cathepsin D in SOD1^G93^ mice. **(G–L)** Immunostaining for cathepsin D counterstained with propidium iodide (PI) of SN-SGC units from control **(G–I)** and SOD1^G93^ mouse **(J–L)**. Note the few immunostained lysosomes in control SGCs and their prominent storage in SGCs from the SOD1^G93^ mouse (insets). Conversely, there are no appreciable differences in the distribution of lysosomes between the control and SOD1^G93^ sensory neurons. Scale bar: **(A,B)** 10 μm; **(G–L)** 5 μm. Student *t*-test in **(C–E)** *p* < 0.001, ***p < 0.001.

We stained semithin sections of DRG with toluidine blue and performed an electron microscopy analysis to determine the morphological alterations induced by the overexpression of mutant SOD1^G93A^ in SN-SGC units. In control SN-SGC units, toluidine blue staining revealed the typical distribution of large (type A), medium (type B), and small size (type C) SN-SGC units in the endoneural tissue microenvironment (Figure 4A). In SOD1^G93A^ mice, the global structural organization of SN-SGC units was preserved at the presymptomatic stage (75 days of age). Intriguingly, numerous SGCs, particularly SGCs from small- and medium-sized units (types B and C), showed a substantial accumulation of basophilic granules, which were identified as lysosomes, in extensive areas of cytoplasm (Figure 4B). Interestingly, when it occurred, the expansion of the lysosomal compartment was frequently observed in several SGCs of the same SN-SGC unit. Abnormal lysosomal storage was rarely observed in SGCs from control DRG (Figure 4A). The proportion of SN-SGC units exhibiting lysosomal storage defect in glial cells, which was estimated using toluidine blue-stained semithin sections, was ~30 and 33% in SOD1^G93A^ mice at 75 and 95 days of age, respectively (Figure 4C). Specifically, this proportion was significantly higher in type B and C units than in larger type A units at both ages (Figures 4D,E).

The lysosomal nature of the basophilic granules was confirmed in dissociated SN-SGC units immunostained for the proteolytic enzyme cathepsin D, a lysosomal marker, and counterstained with propidium iodide. A few cathepsin D-positive lysosomes were observed in control SGCs, whereas large clusters of immunostained lysosomes were frequently observed in SGCs from SOD1^G93A^ mice (Figures 4G–L). Lysosomes were preferentially distributed in the perinuclear cytoplasm in sensory neurons from in both control and SOD1^G93A^ mice, but signs of aberrant lysosomal storage were not detected (Figures 4G–L). A western blot analysis confirmed the increase in levels of the cathepsin D protein in DRG lysates from SOD1^G93A^ mice compared with wild-type animals (Figure 4F).

**Figure 5:**
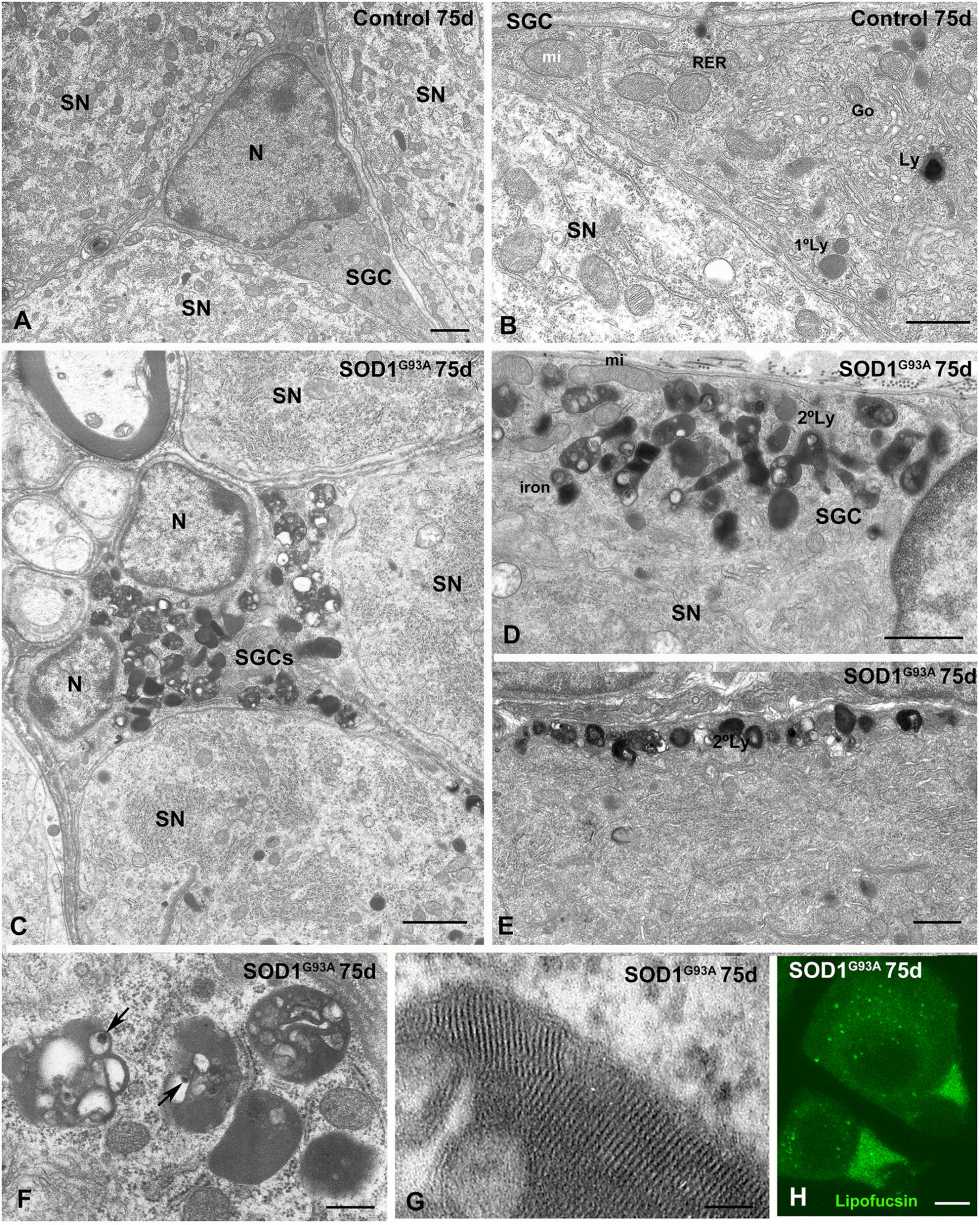
Lysosomal storage disorder in SGCs from SOD1^G93^ mouse at presymptomatic stage (75 days of age). **(A,B)** Ultrastructure of control SGSs surrounding sensory neurons (SN). Note in the **(B)** the characteristic organization of cytoplasmic organelles, including the Golgi complex (Go), endoplasmic reticulum, mitochondria, and some scattered lysosomes (Ly). N: nucleus. **(C)** Panoramic vision of a SGC from a SOD1^G93A^ mouse surrounding three sensory neurons (SN). Note the prominent storage of lysosome-related structures with an heterogenous morphology. **(D,E)** Detail of the focal accumulation of lysosomes at the marginal cytoplasm **(D)** and in a glial expansion **(E)** in SGCs from the SOD1^G93^ mouse. Many secondary lysosomes include electron-lucent areas. **(F)** High magnification of characteristic secondary lysosomes and residual bodies (lipofuscin bodies) containing material of diverse nature including electron-lucent areas and very electron-dense particles (arrows). **(G)** Detail of the intra-lysosomal organizations of a lamellar myelin-like structure. **(H)** Lipofuscin autofluorescence of SN-SGC units from SOD1^G93^ mouse. Note some lipofuscin granules in the cytoplasm of sensory neurons and the higher autofluorescent signal intensity in the cytoplasm of SGCs. Scale bar: **(A–E)** 1 μm; **(F)** 400 nm; **(G)** 50 nm; **(H)** 10 μm.

A low-magnification electron microscopy analysis revealed the morphological organization of SN-SGC units in the DRG from control and SOD1^G93A^ mice at 75 days of age (Supplementary Figures 1A,B). This electron microscopy examination confirmed the presence of SGCs with a lysosomal storage defect (Supplementary Figure 1A). At a higher magnification, the ultrastructural features of SGCs from control DRG were characterized by a cell nucleus with a peripheral distribution of heterochromatin and a perinuclear cytoplasm enriched in mitochondria and Golgi complexes. The cytoplasm also exhibited some endoplasmic reticulum cisternae and isolated lysosomes (Figures 5A,B). Moreover, cell bodies and organelle-poor flat processes of SGCs completely wrapped neuronal perikaryal as previously reported (Pannese, 1981; Hanani, 2005) (Figures 5A,B). In contrast, at the presymptomatic stage, SGCs from the SOD1^G93A^ mice frequently showed an unstructured perinuclear cytoplasm due to the extensive accumulation of densely packed electron-dense bodies identified as lysosome-related structures (Figures 5C,D). Although the lysosomal storage defect preferentially appeared in SGC bodies, numerous lysosomes were also distributed in some flattened processes (Figure 5E). The aberrant accumulation of electron-dense bodies appears to reflect disrupted lysosomal homeostasis (cacostasis), which leads to a non-inherited lysosomal storage pathology in these cells. Abnormal lysosomal storage was accompanied by reductions in both the number of Golgi complexes and protein synthesis machinery, particularly free polyribosomes and ER cisterns (Figures 5C,D).

The storage lysosomes displayed a variety of ultrastructural morphologies. They included small round primary lysosomes with a homogeneous texture and larger polymorphic secondary lysosomes that varied in size or morphology (Figures 5C–E). Some large and complex structures contained intralysosomal (i) electron-lucent areas; (ii) domains with a multilamellar (myelin-like) configuration composed of closely packed lamellae; and (iii) bodies or particles with a very high electron density (Figures 5C,F,G). Regarding the latter, the highly electron dense structures may represent focal deposits of iron or other metals derived from the intralysosomal oxidation of metalloproteases (Figure 5F). Moreover, some complex lysosome-related structures containing large electron-lucent areas may be residual bodies of lipofuscin containing indigestible oxidized lipids and proteins (Figure 5F). The presence of residual bodies of lipofuscin was confirmed by their strong autofluorescence signal detected using confocal microscopy (Figure 5H), a typical feature of lipofuscin granules (Luzio et al., 2007). Moreover, a diffuse cytoplasmic pool of lipofuscin was also observed (Figure 5H).

**Figure 6:**
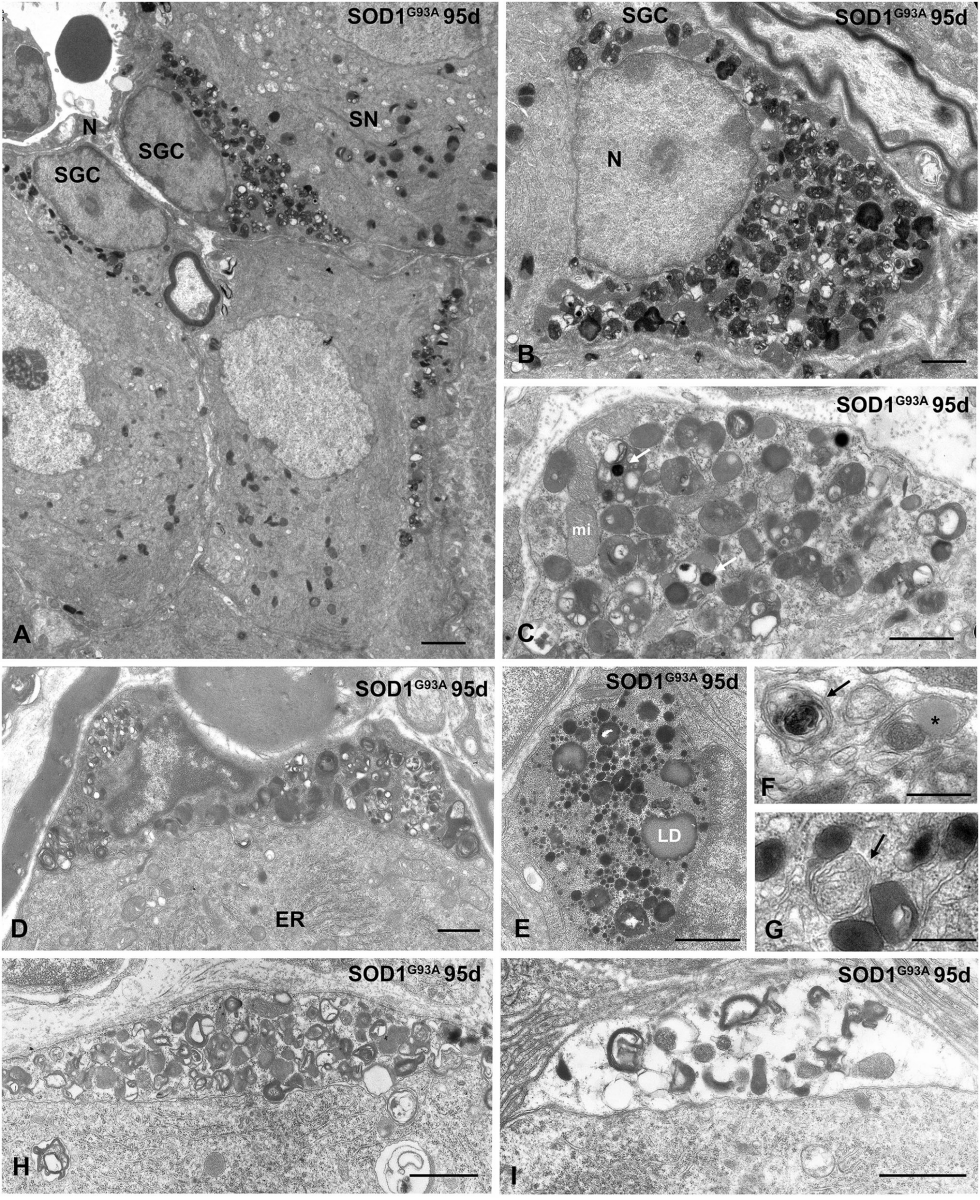
Advanced lysosomal storage disorder in SGCs from SOD1^G93^ mice at symptomatic stage (95 days of age). **(A)** Representative example of several SGSs from a SOD1^G93^ mouse with lysosomal storage. One of the associated sensory neurons (SN) shows abundance of lysosomes and cytoplasmic vacuolation of mitochondria. N: nucleus. **(B)** Massive storage of densely packed lysosomes and residual bodies in the cytoplasm of a SGC with severe disruption of the cytoplasmic organization. **(C)** Storage of lysosomes in a SGC. Note the presence of very electron-dense intralysosomal structures (white arrow) presumably of metal nature and some mitochondria (mi). **(D)** A SGC with high accumulation of lysosomes and prominent heterochromatinization at the nuclear periphery. Note the well-preserved cytoplasmic organization of the associated sensory neuron with a stack of rough endoplasmic reticulum cisterns (ER). **(E)** Large cytoplasmic area with high concentration of small rounded and homogenous primary lysosomes, larger and heterogenous secondary lysosomes, and lipid droplets (LD). **(F,G)** Detail of SGC cytoplasm showing autophagosomes (arrows) and a lipid droplet (asterisk in **F**). **(H,I)** Two progressive stages of vacuolar degeneration in SGCs. Note the complete disruption of glial cell cytoplasm in **(I)**. Scale bar: **(A)** 2 μm; **(B–E)**, **(H,I)** 1 μm; **(F,G)** 0.5 μm.

During the symptomatic stage (95 days of age), the cytoplasmic accumulation of components of the lysosomal system was increased, and some SGCs exhibited signs of advanced cell degeneration, including vacuolar degeneration of lysosomes and other organelles as well as cytoplasmic swelling (Figures 6A–I). Intralysosomal highly electron-dense bodies that were presumably enriched in metals, autophagosomes containing heterogeneous cellular structures and lipid droplets were frequently observed (Figures 6C,E–G). At this symptomatic stage, the SGC alterations were commonly accompanied by an increase in the size of lysosomal compartment and occasional mitochondrial vacuolization in sensory neurons of the SN-SGC units containing SGCs with a lysosomal storage defect (Figure 6A). Based on this finding, SGC degeneration may lead to the metabolic dysfunction of sensory neurons, which specifically affects lysosomal homeostasis.

## Discussion

The present study strengthens the current conception of ALS as a predominant but not an exclusively motor circumscribed disease in which other neuronal and non-neuronal systems may be affected. In our study, the employed behavioral tests mainly evaluate protopathic manifestations, the stimuli of which are principally transmitted by type B and C sensory neurons. Thus, we particularly focused on these neurons and their associated SGCs in the present study.

Interestingly we observed the presence of subtle sensory manifestations even before of the appearance of motor symptomatology. Consistent with findings reported in a previous study by Vaughan et al. (2015) examining two strains of transgenic mice harboring SOD1^G93A^ and TARDBP^A315T^ mutations, the results from the present study suggest that the degeneration of other non-motor areas might precede and/or contribute to the MN damage in ALS. Therefore, at the spinal cord level, complex sensory-motor networks are altered in patients with ALS (Held et al., 2019). Therefore, some degree of involvement of the sensory system may easily contribute to MN damage through different mechanisms, including prion-like propagation (Prusiner, 2012). Previous studies have reported disorders at different levels of the sensory pathway including peripheral receptors, small intraepidermal sensory fibers, sensory neurons in the DRG, anterolateral and dorsal ascending spinal tracts and the sensory cortex (Guo et al., 2009; Vaughan et al., 2015; Rubio et al., 2016; Sassone et al., 2016). However, none of these studies had previously reported SGCs as potential disease targets. Based on the increasing importance of neuron-glia interactions in ALS, the neuron-SGC functional units of DRG have emerged as a pure system to assess not only the pathogenic events but also the chronology of the process due to the particularities of this “functional ecosystem.”

Regarding SOD1 expression in SGCs, our results indicate that the overexpression of mutant SOD1^G93A^ leads to its intracellular accumulation in SGCs of the DRG at presymptomatic motor stages. We propose that the abnormal cytoplasmic accumulation of mutant SOD1^G93A^ in SGCs is potentially neurotoxic by itself and may contribute to the dysfunction of SN-SGC units and the subsequent primary sensory alterations reported here.

In addition to the motor cortex and spinal cord, the abnormal accumulation of mutant SOD1 has also been reported in other brain regions, including the temporal cortex, hippocampus, and cerebellum (Steinacker et al., 2014) as well as DRG neurons in individuals with ALS (Sábado et al., 2014). Importantly, to the best of our knowledge, this is the first study suggesting SGCs as a potential non-motor target in ALS. Several reports have provided evidence that the accumulation of mutant SOD1 in MNs from animal models of ALS leads to altered proteostasis (Riancho et al., 2015). In fact, in SOD1^G93A^ mice, we observed disruptions in proteostasis accompanied by abnormal SOD1 accumulation, the aggregation of ubiquitylated proteins and alterations in the autophagy-lysosomal system in SGCs during the presymptomatic stage. Interestingly, the SOD1^G93A^ mutation has been reported to destabilize and promote protein misfolding and its subsequent aggregation (Sibilla and Bertolotti, 2017). Furthermore mutant SOD1 displays an increased aggregation propensity compared with the wild-type protein (Prudencio et al., 2009). The aggregation of the mutant SOD1 protein may be increased by its ability to propagate its misfolded conformation by acting as a prion-like protein that escapes protein quality control and alters the native SOD1 folding process (Münch and Bertolotti, 2011; Prusiner, 2012; Ayers et al., 2014; Sibilla and Bertolotti, 2017).

Our results are consistent with the induction of oxidative stress in SGCs. Aberrant oxidative reactions catalyzed by mutant SOD^G93A^ have been proposed to contribute to increased oxidative stress and cellular toxicity in ALS (Andrus et al., 2002; Ilieva et al., 2007; Barber and Shaw, 2010; An et al., 2014). In this context, the results of our experiment using the BODIPY-C11 probe revealed increased lipid peroxidation in SGCs of the DRG from SOD1^G93A^ mice. Moreover, lipid peroxidation generates several highly reactive oxidizing agents, including lipofuscin, which are capable of damaging macromolecules and cellular organelles (Höhn and Grune, 2013). Thus, we propose that SGCs of the DRG are important targets of oxidative stress in individuals with ALS.

Notably, lipid droplets composed of neutral lipids were observed in SGCs from the SOD1^G93A^ mice, as evidenced by the BODIPY-493-503 probe and confirmed with electron microscopy. In addition to their essential role in energy storage, growing evidence also links lipid droplets to neuron-glia metabolic coupling (Pennetta and Welte, 2018). In this context, the high level of lipid peroxidation reported here might represent a protective detoxification mechanism for the oxidative stress in sensory neurons. This protective function is relevant to ALS because metabolic abnormalities in lipid metabolism and the lipidome are prevalent in the spinal cord of patients with ALS and SOD1 mice (Chaves-Filho et al., 2019). Moreover, recent evidence suggests a metabolic switch from glucose to lipids as the energy source in SOD1 mouse models of ALS (Schmitt et al., 2014). If this process also occurred in the DRG from the SOD1 mice, neurons would likely rely on SGCs to store excess neutral lipids in droplets as a neuroprotective mechanism by reducing lipotoxicity (Liu et al., 2015; Chaves-Filho et al., 2019). Interestingly, we detected some lipid droplets inside autolysosomes, suggesting a mechanism of lipophagy (Welte and Gould, 2017). Consistently, Rudnick et al. (2017) observed the activation of autophagy in MNs during the progression of ALS in SOD1^G93A^ mice, and we often detected autophagosomes in SGCs from this mouse model of ALS.

The most prominent cellular change observed in SGCs from the SOD1^G93A^ mouse model of ALS is the substantial accumulation of cathepsin D-positive lysosomes and residual bodies, including lipofuscin granules. This cellular response likely substantially affects SGC function and preferentially occurs in types B and C SN-SGC units, consistent with the tactile and thermal-related nociceptive dysfunctions observed in the sensory tests. To the best of our knowledge, our study provides the first observation of the involvement of a lysosomal storage disorder in ALS pathogenesis. Moreover, altered autophagy-lysosomal homeostasis appears to be a major subcellular pathway underlying the toxicity of mutant SOD1^G93A^ toxicity in SGCs. Importantly, increased lysosomal storage in SGCs occurs at the presymptomatic stage (75 days of age) in the SOD1^G93A^ mouse model of ALS, a time prior to the appearance of detectable cellular alterations in sensory neurons. This finding supports the hypothesis that SGCs from the DRG are also primary cellular targets of oxidative stress and proteostasis disturbances in ALS (Supplementary Figure 2). Moreover, the dysfunction of the autophagy-lysosomal system was noted in several SGCs of the same SN-SGC unit, suggesting that the prion-like nature of the mutant SOD1 protein may potentially contribute to the propagation of its toxicity through intercellular communication mechanisms.

We postulate that the pathogenic cascade that leads to excessive storage of lysosomes in SGC is triggered by oxidative stress induced by the abnormal accumulation and toxicity of the mutant SOD1^G93A^ protein. Regarding the potential defects in the activity of lysosomal enzymes in SGCs, aberrant changes in glucosylceramide, galactolipids, and sphingomyelin levels have been reported in the spinal cord of patients with ALS and SOD1 mouse models of the disease (Dodge et al., 2015).

The lysosomal storage disorder in SGCs appears to reflect a severe disturbance of proteostasis that particularly affects cellular mechanisms of macromolecular degradation. In this context, dysfunction of both the ubiquitin proteasome and autophagy-lysosomal systems has been reported in the spinal cord of patients with ALS and SOD1^G93A^ mice (Rudnick et al., 2017). Consistent with this observation, we observed an abundance of autophagosomes in SGCs from this mouse model of ALS.

## Conclusions

Our study strengthens the current widely accepted concept of ALS as a non-MN exclusively circumscribed disorder, supporting the sensory involvement noted in the SOD1 transgenic murine model of the disease. Specifically, we first highlighted SGCs as a new potential target for the disease (Supplementary Figure 2). The preferential lysosomal pathology observed in these cells meets the criteria for a lysosomal storage disorder induced by oxidative stress (Kielian, 2019; Marques and Saftig, 2019). Moreover, SGCs appear to have an essential function in maintaining sensory neurons homeostasis, thus reinforcing the crucial role of astroglial cells in ALS (Baker et al., 2015). Based on both the existence of sensory-motor networks in the spinal cord and the prion hypothesis, SGCs might be responsible for the sensory manifestations and potentiate or propagate motor neuron pathology. The inclusion of these “new guests” into ALS pathogenesis will provide new pathogenic perspectives that might result in the development novel therapeutic strategies.

## Data Availability Statement

The raw data supporting the conclusions of this article will be made available by the authors, without undue reservation.

## Ethics Statement

The animal study was reviewed and approved by COMITE DE ÉTICA DE LA UNIVERSIDAD DE CANTABRIA.

## Author Contributions

JR, ML, and MB designed and supervised the experiments and wrote the manuscript. MR-S, JR, OT, and MB performed the experiments and analyze the data. MR-S, JR, OT, MB, and ML read and editing the article. All authors contributed to the article and approved the submitted version.

## Conflict of Interest

The authors declare that the research was conducted in the absence of any commercial or financial relationships that could be construed as a potential conflict of interest.
