# Supplementary material for: Satellite Glial Cells of the Dorsal Root Ganglion: A New “Guest/Physiopathological Target” in ALS
Source: Front Aging Neurosci. 2020 Nov 9;12:595751. doi: 10.3389/fnagi.2020.595751 (PMC7680735; doi:10.3389/fnagi.2020.595751)
Supplement: Supplementary file 1 [file Table_1.DOCX]

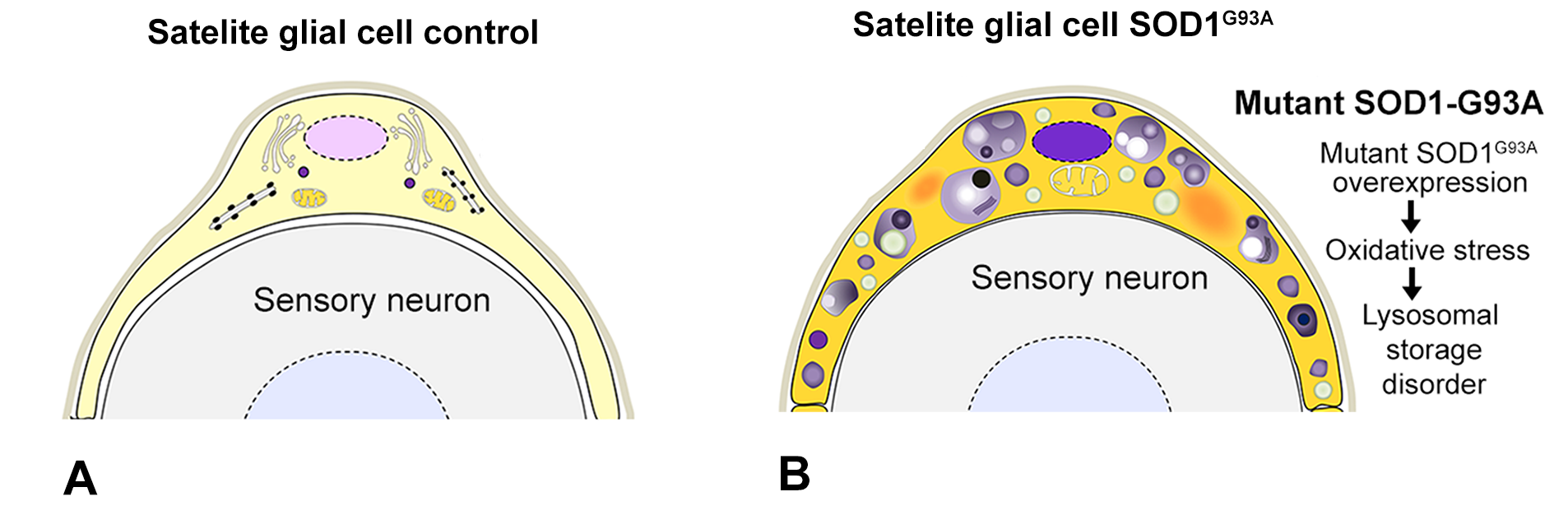
**Figure S1**. (**A, B**) Panoramic view of SN-SGC units from control (A) and SOD1^G93^ mice (B) at 75 days of age. SN: sensory neuron; SGC: satellite glial cell. Note the accumulation of lysosomes in SGCs from the SOD1^G93^ mice (white arrows). Scale bars: A, B 10µm
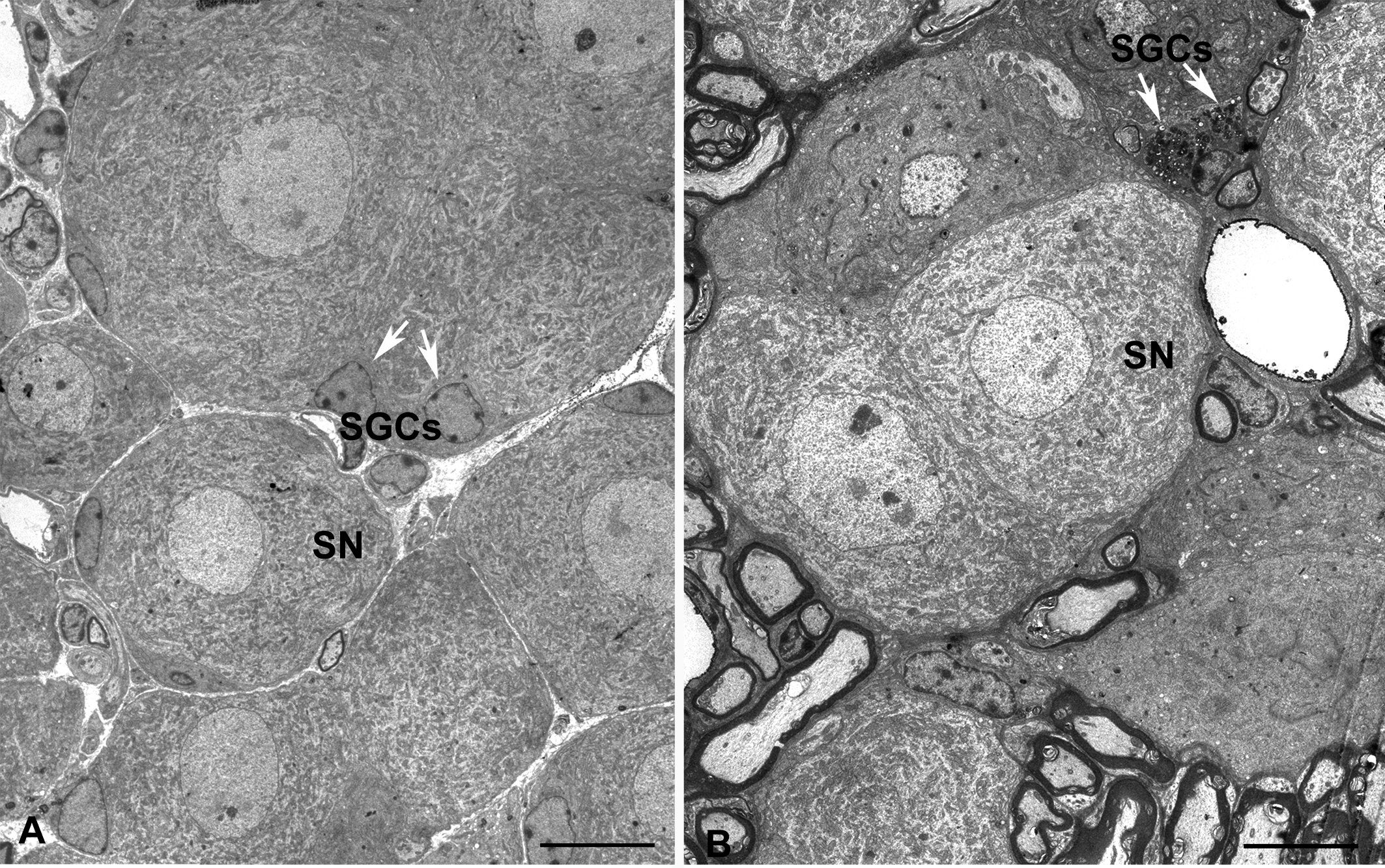


**Figure S2**. Satellite glial cells (SGCs) of the dorsal root ganglia emerge as a new pathophysiological ltarget in the SOD1^G93A^ mouse model of ALS. Overexpression of the mutant SOD1-G93A induces oxidative stress in SGCs resulting in a lysosomal storage disorder and ultimately glial cell degeneration
